# Prognostic Value of Tumor-Associated Macrophages in Clear Cell Renal Cell Carcinoma: A Systematic Review and Meta-Analysis

**DOI:** 10.3389/fonc.2021.657318

**Published:** 2021-04-26

**Authors:** Haixiang Shen, Jin Liu, Shiming Chen, Xueyou Ma, Yufan Ying, Jiangfeng Li, Weiyu Wang, Xiao Wang, Liping Xie

**Affiliations:** ^1^ Department of Urology, First Affiliated Hospital, Zhejiang University School of Medicine, Hangzhou, China; ^2^ Department of Surgical Oncology, First Affiliated Hospital, Zhejiang University School of Medicine, Hangzhou, China

**Keywords:** tumor-associated macrophages, clinicopathological significance, survival, clear cell renal cell carcinoma, biomarker

## Abstract

**Background:**

Tumor-associated macrophages (TAMs) are the major immune cells in tumor microenvironment. The prognostic significance of TAMs has been confirmed in various tumors. However, whether TAMs can be prognostic factors in clear cell renal cell carcinoma (ccRCC) is unclear. In this study, we aimed to clarify the prognostic value of TAMs in ccRCC.

**Methods:**

We searched PubMed, Embase, and the Web of Science for relevant published studies before December 19, 2020. Evidence from enrolled studies were pooled and analyzed by a meta-analysis. Hazard ratios (HRs) and odd ratios (ORs) with 95% confidence intervals (CIs) were computed to evaluate the pooled results.

**Results:**

Both of high CD68+ TAMs and M2-TAMs were risk factors for poor prognosis in ccRCC patients. The pooled HRs indicated that elevated CD68+ TAMs correlated with poor OS and PFS (HR: 3.97, 95% CI 1.39–11.39; HR: 5.73, 95% CI 2.36–13.90, respectively). For M2-TAMs, the pooled results showed ccRCC patients with high M2-TAMs suffered a worse OS and shorter PFS, with HR 1.32 (95% CI 1.16–1.50) and 1.40 (95% CI 1.14–1.72), respectively. Also, high density of TAMs was associated with advanced clinicopathological features in ccRCC.

**Conclusions:**

TAMs could be potential biomarkers for prognosis and novel targets for immunotherapy in ccRCC. Further researches are warranted to validate our results.

## Introduction

Renal cell carcinoma (RCC) is a major malignancy in human urinary system, in which clear cell renal cell carcinoma (ccRCC) is the most common histologic type. Globally, it was reported that RCC accounted for about 2.2% of all tumors with approximately 403,262 new cases in 2018, and the mortality was around 175,098 (1.8%) (1). The incidence or mortality profile of RCC was associated with human development index (HDI) of regions. People from high/very high HDI countries had a higher incidence and mortality of RCC in both male and female than whom in low/medium HDI regions (1). The overall survival (OS) of RCC has been improved because of early detection and more efficient treatment including radical, partial nephrectomy and targeted therapy (2). However, the survival outcome for advanced or metastatic RCC is still poor (3, 4).

Tumor-associated macrophages (TAMs), as a major group of immune cells in tumor microenvironment (TME), were reported playing a critical role in tumor development (5). Two main phenotypes of TAMs—M1 and M2, are activated by different cytokines and chemokines (6–8). M1-TAMs were considered to be responsible for antigen presentation and inflammation, while M2-TAMs were believed participating in tumorigenesis and progression process like tumor growth, metastasis and treatment resistance (9–11).

Some studies discovered TAMs were associated with survival outcomes in various tumors and uncovered the potential value of TAMs to be novel biomarkers for prognosis and target-therapy (12–15). However, the prognostic role of TAMs in RCC was not yet confirmed. In this meta-analysis, our main purpose was to investigate the prognostic role of TAMs in ccRCC patients. Also, we tried to settle down whether TAMs were associated with the clinicopathological factors in ccRCC.

## Materials and Methods

We conducted out this meta-analysis in accord with the Preferred Reporting Items for Systematic Reviews and Meta-Analyses (PRISMA) statement (16).

### Search Strategy

We retrieved published articles from PubMed, Embase, and the Web of Science before December 19, 2020. The main search terms were as follows: “renal cell carcinoma”, “renal”, “kidney”; “neoplasm”, “carcinoma”, “cancer”, “malignancy”; “macrophage”, “CD68”, “CD86”, “CD163”, “CD169”, “CD204”, “CD206”; “prognosis”, “prognostic”, “survival”, “biomarker”. We also scanned the references of the eligible studies for additional candidate trials that met our inclusion criteria.

### Inclusion and Exclusion Criteria

Two authors (HS and JL) assessed all potentially eligible studies by screening the titles and abstracts, and then identified through full-text reading independently. Any discrepancy between the two authors was solved by discussion or consulting a third author. The inclusion criteria were as follows: 1) the diagnosis of ccRCC was confirmed by histopathological examination; 2) tumor-associated macrophages were evaluated in tumor tissues; 3) the information about clinicopathological characteristics or prognosis of patients was displayed; 4) studies were published in English. Studies that met the exclusion criteria were excluded: 1) no full-text accessible; 2) duplicate studies; 3) animals or cell-line studies without human; 4) reviews, meeting abstracts, expert opinions, letters, editorials, or case reports.

### Evaluation of Study Quality

Two authors (HS and JL) evaluated the quality of selected studies independently by implementing the Newcastle-Ottawa Scale (NOS) (17). We solved the disagreements by discussion. The quality was regarded as high if the score of NOS was ≥7.

### Data Extraction

The data were collected by all authors. 1) The essential information of enrolled studies including the name of first author, country, published year, study type, sample size, TAMs detection assay, dichotomization form, evaluation methods and cut-off values; 2) The patient characteristics included age, gender, tumor stage, nodal status, nuclear necrosis, vascular invasion, nuclear grade, and median follow-up time; 3) Survival data included hazard ratios (HRs) with 95% confidence intervals (CIs) for progression-free survival (PFS), cancer-specific survival (CSS) and OS.

### Statistical Analysis

For evaluating prognostic value of the TAMs in patients with ccRCC, we calculated pooled HRs with 95% CIs for PFS, CSS and OS. For exploring the correlation between TAMs and clinicopathological factors, odd ratios (ORs) with 95% CIs were computed. Patients were divided into two groups by age (>60 *vs* ≤60), gender (male *vs* female), tumor stage (pT3–T4 *vs* pT1–T2), UICC-stage (III–IV *vs* I–II), tumor necrosis (positive *vs* negative) and nuclear grade (G3–4 *vs* G1–2). We identified statistical heterogeneity of different studies *via* employing the Chi-square-based Q statistics and I^2^ value (18). A fixed-effects model was applied when I^2^ >50% or *p <*0.10. Otherwise, a random-effects model was implemented. We carried out sensitivity analysis to evaluate the stability of pooled results and used Begg’s test to determine the potential publication bias among included studies. All the statistical analyses were realized by STATA software (version 12.0, Stata Corp LP, TX77845, USA). Two-tailed *p*-value <0.05 was regard as statistical significance.

## Results

### Search Results and Study Features

**Figure 1:**
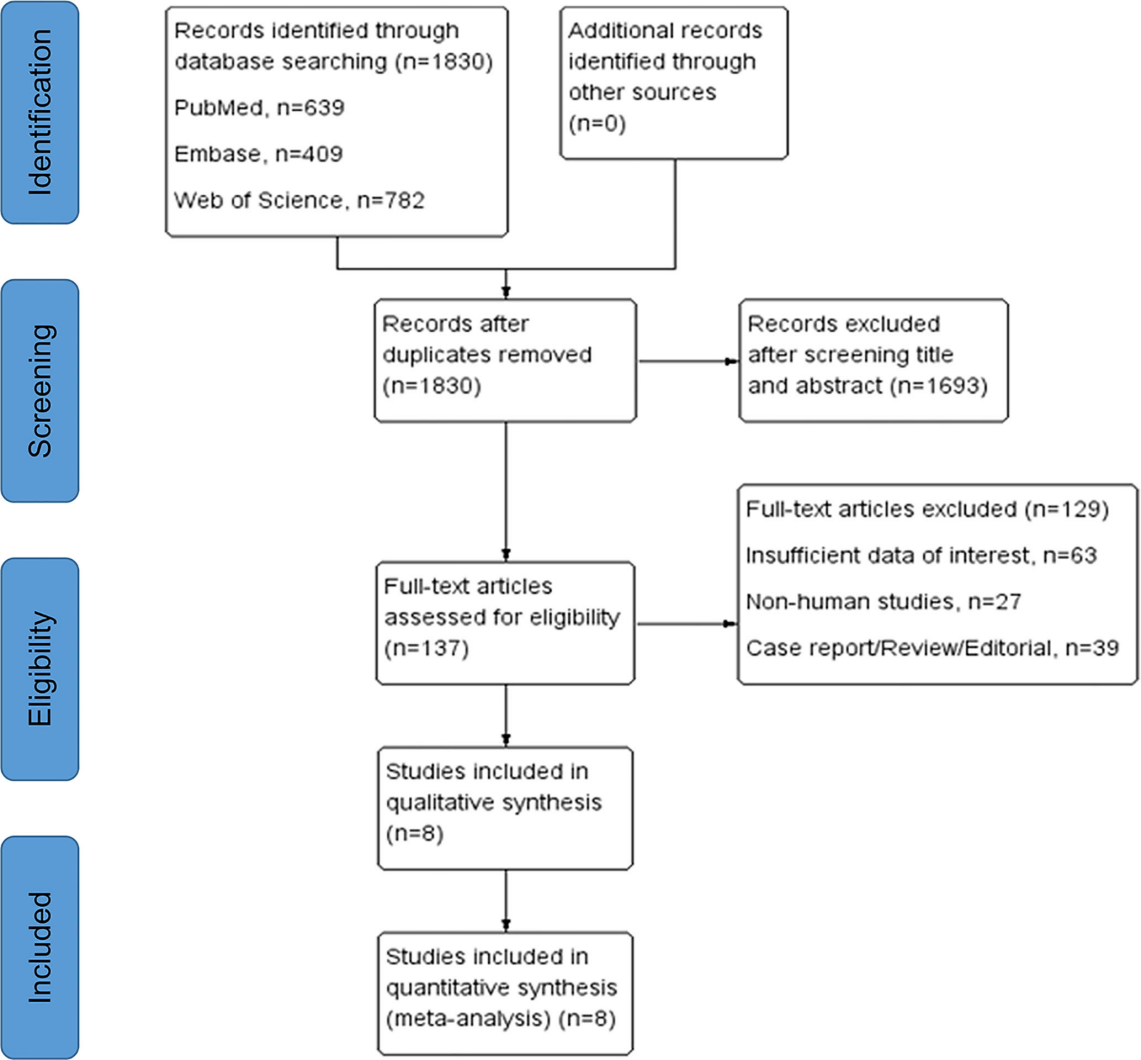
PRISMA flowchart for study selection process.

We included eight studies with total 1,122 patients that fulfilled our inclusion criteria in this research. The detailed selection process was displayed in **Figure 1**. According to the NOS, the quality of included studies was high (**Supplementary Table 2**). All the eligible studies were published from 2011 to 2020, with the sample size ranging from 54 to 257. Among them, four studies were conducted in Japan (19–22), two in China (23, 24), one in Switzerland (25) and one in France (26). Immunohistochemistry assay was applied to TAMs detection except one study using quantitative polymerase chain reaction (25). In terms of biomarker for TAMs evaluation, CD68 was used in five studies, CD163 in five, CD204 in two, CD206 and CD11c in one. PFS, CSS, and OS were reported as prognostic endpoints in four, two and five studies, respectively. **Table 1** shows the main characteristics of included studies and the more detailed information about target specimens, assays, dichotomization forms and the cutoff values is presented in **Supplementary Table 1**.

**Table 1:** Characteristics of studies included in the meta-analysis.

Study	Country	TAM detection assay	TAM marker		Patient characteristics							
				Tumor status	Patients, n	Age, yearmean ± SD (range)	Gender, n (male/female)	Tumor status, n (%)	Fuhrman grade, n (%)	Tumor necrosis, n (%)	Tumor grade, n (%)	Median follow-up, month (range)
Komohara et al. (19)	Japan	IHC	CD163	Primary ccRCC	66	NA	46/20	T1, 30 (45.5); T2,3, 36 (54.5)	G1 10 (15.2), G2 32 (48.5), G3,4 24 (36.3)	NA	G1,2 42 (63.6); G3,4 24 (36.4)	NA
Dannenmann et al. (25)	Switzerland	qPCR	CD68, CD163	Primary ccRCC with no prior treatment	54	66.3 ± 7.2 (40–86)	NA	T1, 21 (38.9); T2, 4 (7.4); T3, 28 (51.9); T4, 1 (1.8)	NA	NA	NA	80.9 ± 64.2^‡^ (NA)
Xu et al. (23)	China	IHC	CD68, CD11c, CD206	Primary ccRCC with no prior treatment	185	60.7 ± 12.4 (30–84)	70/115	T1, 119 (64.3); T2, 33 (17.9); T3, 33 (17.9)	G1,2, 74 (40.0%) G3,4, 111 (60.0%)	Absent 106(57.3), present 79(42.7)	NA	70 (10–120)
Komohara et al. (20)	Japan	IHC	CD204	Primary ccRCC undergone curative surgery	91	NA	59/32	T1, 42 (46.2); T2–4, 49 (53.8);	G1,2 74 (81.3), G3,4 17 (18.7)	NA	NA	NA
Cros et al. (26)	France	IHC	CD68, CD163	Primary ccRCC	257	61.3 ± 11.7 (NA)	171/86	T1 143 (55.6), T2 26 (10.1), T3 85 (33.1), T4 3 (1.2)	NA	NA	G1,2 156 (60.7), G3,4101 (39.3)	72 (22.8–112.8)
Nakanishi et al. (21)	Japan	IHC	CD68	Primary ccRCC with no prior treatment	179	NA	NA	NA	NA	NA	NA	39 (19–62)^§^
Ma, 2017	Japan	IHC	CD163	Primary ccRCC undergone curative surgery	103	NA	54/49	T1, 66 (64.1); T2–4, 37 (35.9)	G1,2 81 (78.6), G3,4 22 (21.4)	NA	NA	NA
Wang et al. (24) cohort 1	China	IHC	CD68, CD163	Primary ccRCC	110	NA	87/23	NA	NA	NA	G1,2 68 (61.8), G3,4 42 (38.2)	NA
Wang et al. (24) cohort 2	China	IHC	CD68, CD163	Primary ccRCC	143	NA	85/58	NA	NA	NA	G1,2 107 (74.8), G3,4 36 (25.2)	NA

TAM, tumor-associated macrophage; IHC, immunohistochemistry; ccRCC, clear cell renal cell carcinoma, qPCR, quantitative polymerase chain reaction; NA, not available.

^‡^Mean ± Standard deviation.

^§^Median (Interquartile range).

### TAMs and Prognosis in ccRCC

#### CD68+ TAMs Prognostic Significance in ccRCC

**Figure 2:**
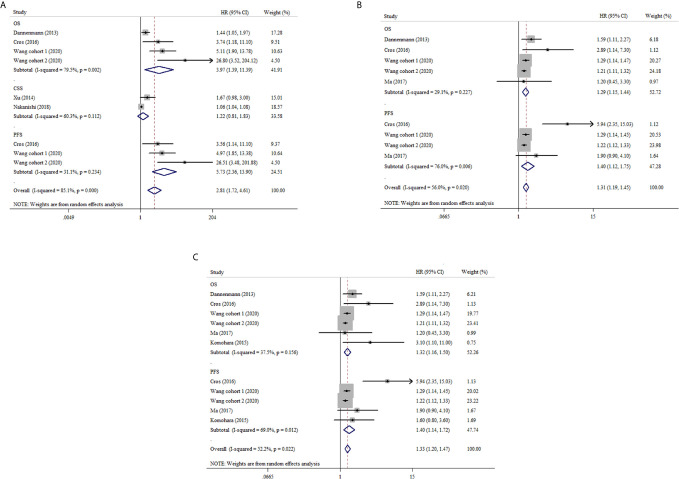
Forest plots of HRs for prognostic value with high versus low density of TAMs in RCC patients. **(A)** HRs of OS, CSS and PFS for CD68+ TAMs in RCC; **(B)** HRs of OS and PFS for CD163+ TAMs in RCC; **(C)** HRs of OS and PFS for M2-TAMs in RCC.

To investigate the prognostic significance of CD68+ TAMs in ccRCC, we calculated the pooled HRs for OS, CSS and PFS. **Figure 2A** and **Supplementary Table 3** show that the pooled HR is 3.97 with 95% CI 1.39–11.39 (I^2^ = 79.5%, *p* = 0.002) for OS, which demonstrates higher CD68+ TAMs predicting worse OS. The pooled HR for PFS indicated that elevated CD68+ TAMs was correlated with poor PFS (HR: 5.73, 95% CI 2.36–13.90, I^2^ = 31.1%, *p* = 0.234). No statistical significance was obtained for the CD68+ TAMs value on CSS (HR: 1.22, 95% CI 0.81–1.83, I^2^ = 60.3%, *p* = 0.112).

#### CD163+ TAMs Prognostic Significance in ccRCC

Four studies reported the prognostic value of CD163+ TAMs in ccRCC. The pooled results demonstrated that high density of CD163+ TAMs was associated with poor OS with HR 1.29 (95% CI 1.15–1.44, I^2^ = 29.1, *p* = 0.227). As regard to PFS, we also observed a similar result from three studies using a random-effect model (HR: 1.40, 95% CI 1.12–1.75, I^2^ = 76.0%, *p* = 0.006) (**Figure 2B** and **Supplementary Table 4**).

#### CD11c+, CD204+ and CD206+ TAMs Prognostic Significance in ccRCC

Two studies uncovered the role of CD11c+, CD 204+ and CD206+ TAMs in survival outcomes of ccRCC, respectively. Xu et al. reported that macrophages marked by CD11c showed a protective factor on CSS in ccRCC (HR: 0.48, 95% CI 0.23–1.00, *p* = 0.049), however, CD206+ TAMs played an opposite role (HR: 1.95, 95% CI 1.11–3.42) (23). From another research, Komohara et al. illustrated that high density of CD204+ TAMs was an adverse factor for OS (HR: 3.1, 95% CI 1.10–11.00), while no statistical significance was found for PFS (HR: 1.6, 95% CI 0.8–3.6) (20). We did not conduct meta-analysis on the above markers of TAMs in prognosis because of few data.

#### M2-TAMs Prognostic Significance in ccRCC

In our meta-analysis, CD163+, CD204+ and CD206+ TAMs were regard as M2-TAMs. The pooled analysis indicated that M2-TAMs had a negative correlation with OS in ccRCC (HR: 1.32, 95% CI 1.16–1.50, I^2^ = 37.5%, *p* = 0.156). Also, the high density of M2-TAMs was associated with poor PFS in ccRCC (HR: 1.40, 95% CI 1.14–1.72, I^2^ = 69.0%, *p* = 0.012) (**Figure 2C** and **Supplementary Table 5**).

### TAMs and Clinicopathological Features in ccRCC

#### CD68+ and CD163+ TAMs

**Figure 3:**
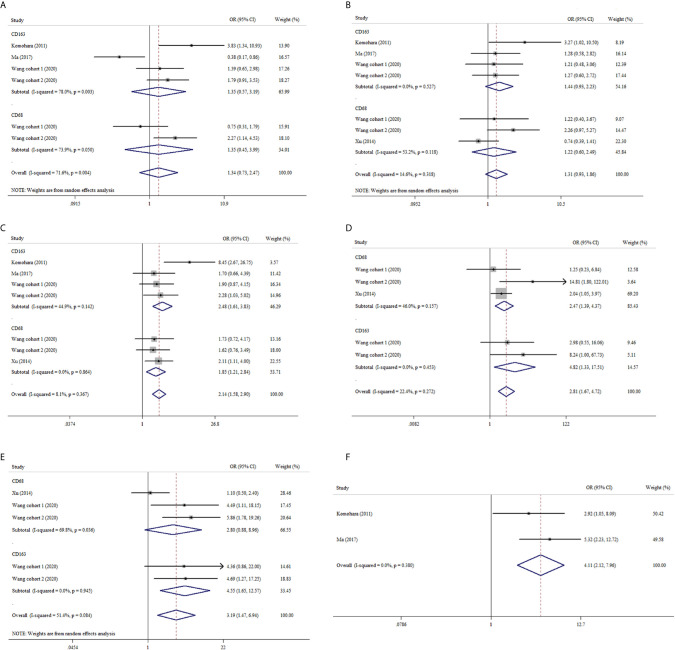
Forest plots of ORs accessing the correlation between CD68+ or CD163+ TAMs density and clinicopathological features. **(A)** CD68+ or CD163+ TAMs and age; **(B)** CD68+ or CD163+ TAMs and gender; **(C)** CD68+ or CD163+ TAMs and nuclear grade; **(D)** CD68+ or CD163+ TAMs and tumor necrosis; **(E)** CD68+ or CD163+ TAMs and UICC-stage; **(F)** CD163+ TAMs and tumor stage.

We also analyzed the association between TAMs and clinicopathological features in ccRCC. Our pooled analysis suggested that no significant relationship between CD68+ or CD163+ TAMs and age or gender in ccRCC (**Figures 3A, B**). As shown in **Figure 3C**, both CD68+ TAMs and CD163+ TAMs were associated with higher nuclear grade (OR: 1.85, 95% CI 1.21–2.84, I^2^ = 0.0%, *p* = 0.864; OR: 2.48, 95% CI 1.61–3.83, I^2^ = 44.9%, *p* = 0.142). From **Figure 3D**, we also found tumor necrosis were more likely to present in ccRCC with increased CD68+ or CD163+ TAMs (OR: 2.47, 95% CI 1.39–4.37, I^2^ = 46.0%, *p* = 0.157; OR: 4.82, 95% CI 1.33–17.51, I^2^ = 0.0%, *p* = 0.453, respectively). Furthermore, ccRCC patients with high CD163+ TAMs showed a trend of advanced UICC-stage (OR 4.55, 95% CI 1.65–12.57, I^2^ = 0.0%, p = 0.084) while CD68+ TAMs did not (OR: 2.80, 95% CI 0.88–8.96, I^2^ = 69.8%, *p* = 0.036) (**Figure 3E**). In addition, pooled results from two studies indicated that elevated CD163+ TAMs were more prone to advanced tumor-stage with a fixed-effects model (OR: 4.11, 95% CI 2.12–7.96) (**Figure 3F**).

#### M2-TAMs

**Figure 4:**
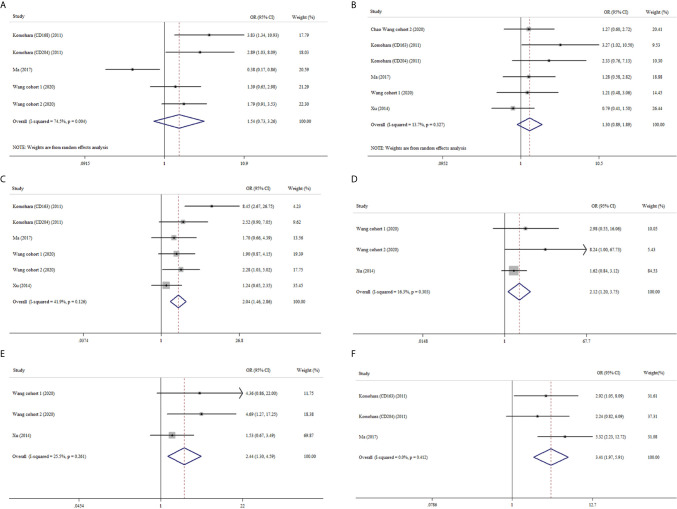
Forest plots of ORs accessing the correlation between M2-TAMs density and clinicopathological features. **(A)** M2-TAMs and age; **(B)** M2-TAMs and gender; **(C)** M2-TAMs and nuclear grade; **(D)** M2-TAMs TAMs and tumor necrosis; **(E)** M2-TAMs TAMs and UICC-stage; **(F)** M2-TAMs TAMs and tumor stage.

Similarly, M2-TAMs had no significant correlation with age or gender (OR: 1.54, 95% CI 0.73–3.26; OR: 1.30, 95% CI 0.89–1.89) (**Figures 4A, B**). Four studies provided relative data about M2-TAMs and nuclear grade of ccRCC (19, 22–24). The meta-analysis results delineated M2-TAMs preferred to exist in ccRCC with more advanced nuclear grade (OR: 2.04, 95% CI 1.46–2.86, I^2^ = 41.9%, *p* = 0.126) (**Figure 4C**). Tumor necrosis were more likely to be observed in ccRCC with high density of M2-TAMs (OR: 2.12, 95% CI 1.20–3.75, I^2^ = 16.3%, *p* = 0.303) (**Figure 4D**). What’s more, we found the prevalence of M2-TAMs was more common in ccRCC with more advanced UICC-stage or tumor-stage (OR: 2.44, 95% CI 1.30–4.59, I^2^ = 25.5%, *p* = 0.261; OR: 3.41, 95% CI 1.97–5.91, I^2^ = 0.0%, *p* = 0.412) (**Figures 4E, F**).

### Sensitivity Analysis

**Figure 5:**
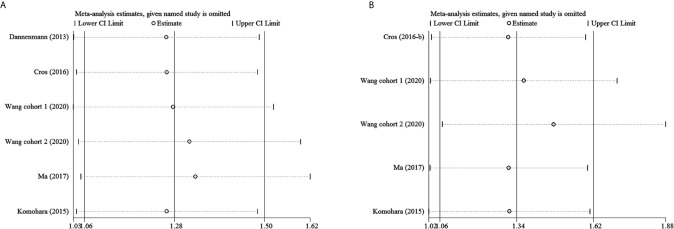
Sensitivity analysis. **(A)** M2-TAMs and OS; **(B)** M2-TAMs and PFS.

To examine the stability of pooled HRs, sensitivity analyses were performed. **Figure 5** and **Supplementary Figure 1** show that the results are stable.

### Publication Bias Assessment

Begg’s test was performed to assess the publication bias of the included studies. The results showed that no significant publication bias was present in the studies (**Supplementary Figures 2**–**4** and **Supplementary Tables 3**–**5**).

## Discussion

Many researches reported that TME played an important role in tumorigenesis, progress and metastasis (27, 28). TAMs, as major complement in TME, have gained increasing attention recently. Previous published studies had illustrated that higher density of TAMs was correlated with worse survival outcomes or advanced clinical features in head and neck squamous cell carcinoma (13), breast cancer (12), non-small-cell lung cancer (29), gastric cancer (30), hepatocellular carcinoma (HCC) (31), pancreatic cancer (32) and ovarian cancer (14). Whereas, in colorectal cancer (CRC), an opposite result was obtained (33, 34). In this study, we performed a meta-analysis on prognostic significance of TAMs in ccRCC. Our results showed that high TAMs predicted poor prognosis. Moreover, we also found high TAMs was more frequent to be seen in ccRCC with advanced clinicopathological features like tumor necrosis, high UICC stage and nuclear grade.

TAMs originate from monocyte circulating in peripheral blood, and are recruited to tumor sites by various receptors that expressed on tumor cells for cytokines derived from macrophages (35, 36). TAMs differentiate into two main phenotypes—M1-TAMs and M2-TAMs under different TME (7). M1-TAMs are induced by lipopolysaccharide and T helper type 1 (Th1) cytokines like interferon-γ (IFN-γ), tumor necrosis factor α (TNF-α), and granulocyte-macrophage colony-stimulating factor (GM-CSF). The major functions of M1-TAMs are antigen presentation, inflammation inducing and tumor preventing (7, 35, 37). Activated by interleukin 3 (IL-3), IL-4, IL-10, IL-13, colony stimulating factor 1 (CSF-1) and prostaglandin E2 (PGE-2), M2-TAMs can inhibit inflammatory response (38–40), induce angiogenesis, and participate in tumorigenesis and progression (7, 41). CD68 is the main macrophage marker used to identify general TAMs including M1- and M2-TAMs, while CD163, CD204 and CD206 are commonly employed for M2-TAMs detection (42–44). For the prognostic role of CD68+ TAMs, the results reported from different studies were inconsistent in various tumors. Some studies demonstrated that high density of CD68+ TAMs was associated with poor prognosis which was similar in our study (12, 32, 45, 46), while Zhao et al. revealed an opposite result in CRC (15). In addition, even no significant correlation between CD68+ TAMs and prognosis was obtained in other studies (13, 14, 31). Also, some studies reported that the survival effects of CD68+ TAMs tested in stroma and tumor islet were opposite (29). In the research of Ohri et al. and Ma et al., they found that the M1/M2 TAMs ratio was different in stroma and tumor islet, which could explain the different prognostic role of CD68+ TAMs marked as general macrophages (47, 48). Xia et al. suggested that M1/M2 TAMs ratio maybe a better biomarker for survival outcome (14). Most studies selected CD163 as biomarker for M2-TAMs detection, only few of them used CD204 and CD206. In our meta-analysis, CD163 was used in five studies (19, 22–25), CD204 (19, 20) in two and CD206 (23) in one, respectively. The pooled results showed that high density of M2-TAMs was a risk factor of poor prognosis in ccRCC. Similar results were obtained in other solid tumors (12–15, 31).

Although, only a few of included studies reported essential information about clinicopathological features, we explored the clinicopathological significance of TAMs. In our meta-analysis, the pooled results showed both high CD68+ TAMs and M2-TAMs were associated with tumor necrosis and advanced nuclear grade. Furthermore, we also found high M2-TAMs were more common to be observed in ccRCC with advanced UICC stage and tumor-stage (T-stage). These findings supported the prognostic value of TAMs in ccRCC.

Recently, accumulated researches have revealed the crucial role of TAMs in tumorigenesis and development, and suggested that TAMs might be potential therapeutic targets. As for development of drug targeting TAMs, some preclinical and clinical trials are currently underway. Germano et al. found that trabectedin could deplete TAMs by activating caspase-8-dependent apoptosis and exert anti-tumor function (49). Some studies demonstrated TAMs participated in epithelial–mesenchymal transition (EMT) and promoted tumor metastasis (50–52). Sorafenib could inhibit macrophage-induced EMT to prevent HCC cells from migration, which was found by Deng et al. (52). What’s more, M1- and M2-TAMs can shift into each other under specific signals in TME (35, 53–55). Therefore, targeting to increase M1/M2 ratio and strengthening the anti-tumor role of M1-TAMs could be a novel strategy for tumor treatment. Through reprograming M2-TAMs toward into M1-TAMs, humanized anti-CD40 antibodies could suppress progression of pancreatic cancer and provide a better prognosis, which was confirmed in mouse models and patients with pancreatic ductal adenocarcinoma (56). Toll-like receptor (TLR) agonists could also remodel the polarization of TAMs from M2- to M1-TAMs, therefore, promote the antitumor immune reaction (57). However, the monotherapy of TLR agonists always results in the compensatory effect of increased expression of PD-L1 in macrophages, which can bind to PD-1 expressed on activated T cells and cause immunosuppression, consequently lead to immune evasion of tumors (58). To overcome this limitation, combination therapy of the TLR agonists in conjunction with PD-1 blockade has been used in cancer treatment and achieved better therapeutic efficacy (59, 60). Inhibitors of the CSF-1/CSF-1 receptor (CSF-1R) could inactivate monocyte progenitors generating and TAMs differentiation, which benefited better survival outcomes (37, 61). Yang et al. identified that Wnt/β-catenin signaling pathway was involved in M2-TAMs polarization and facilitated tumor progression (62). Thus, blockade of this pathway using selective small molecule inhibitors may be a potential strategy to decrease the number of M2-TAMs, which could be a novel adjuvant therapy for patients with tumor. From the preclinical data of Aggen et al., it was reported that anti-IL1β monotherapy or combination therapy with anti-IL1β plus anti-PD-1/cabozantinib inhibited tumor growth significantly in a renal cell carcinoma model through decreasing immunosuppressive myeloid-derived suppressor cells and increasing M1-TAMs in TME (63). Their work indicated that IL1β was a potential novel target for immunotherapy in RCC.

Although trials on TAMs-targeted therapy showed favorable effects on tumor, the crosstalk between TAMs and tumor cells should be further clarified. Further researches are needed to uncover detailed molecular mechanisms of TAMs’ role in TME underlying tumor development.

This is the first meta-analysis that investigates the prognostic role of TAMs in ccRCC. Our pooled results confirmed the potential of TAMs to be biomarkers for prognosis and targets for therapy in ccRCC. However, some limitations in our study should be considered. First, six of eight enrolled studies were conducted in Asia (four in Japan and two in China), which restricted these results to Asian population. Second, the number of eligible studies was relative small and the sample size was not large enough. Because of few studies reported detailed information of clinicopathological features, the association between TAMs and clinicopathological factors was not definite. Third, different antibodies and various antibodies concentrations were applied in enrolled studies, and the inconsistent evaluation methods and cut-off value may lead to heterogeneity. Fourth, no subgroup analysis for prognosis was performed due to the scarcity of enough data. Fifth, only studies published in English language were enrolled which could result in potential publication bias.

## Conclusion

In conclusion, this was the first meta-analysis on the prognostic value of TMAs in ccRCC. Our results revealed that both high density of CD68+ TAMs and M2-TAMs were correlated with poor OS and PFS. Also, our study demonstrated that high density of TAMs was associated with advanced clinicopathological factors. However, due to the limitations in this study, our results should be consulted with cautiousness. Furthermore, large-scale and multiple centers prospective researches are warranted to validate our results.

## Author Contributions

Study concept and design: XW and LX. Acquisition of data: HS, JLiu, SC, XM, YY, WW, and JLi. Analysis and interpretation of data: HS, JLiu, and SC; Statistical analysis: HS and JLiu. Drafting of the manuscript: HS and JLiu. Critical revision of the manuscript for important intellectual content: XW and LX. All authors contributed to the article and approved the submitted version.

## Conflict of Interest

The authors declare that the research was conducted in the absence of any commercial or financial relationships that could be construed as a potential conflict of interest.

## Supplementary Material

Supplementary Figure 1 Sensitivity analysis. **(A)** CD163+ TAMs and OS; **(B)** CD163+ TAMs and PFS.

Supplementary Figure 2 The Begg’s funnel plots of the association between CD68+ TAMs and clinicopathological features or prognosis. **(A)** CD68+ TAMs and age; **(B)** CD68+ TAMs and gender; **(C)** CD68+ TAMs and UICC stage; **(D)** CD68+ TAMs and necrosis; **(E)** CD68+ TAMs and nuclear grade; **(F)** CD68+ TAMs and OS; **(G)** CD68+ TAMs and PFS; **(H)** CD68+ TAMs and CSS.

Supplementary Figure 3 The Begg’s funnel plots of the association between CD163+ TAMs and clinicopathological features or prognosis. **(A)** CD163+ TAMs and age; **(B)** CD163+ TAMs and gender; **(C)** CD163+ TAMs and pT; **(D)** CD163+ TAMs and UICC stage; **(E)** CD163+ TAMs and necrosis; **(F)** CD163+ TAMs and nuclear grade; **(G)** CD163+ TAMs and OS; **(H)** CD163+ TAMs and PFS.

Supplementary Figure 4 The Begg’s funnel plots of the association between M2-TAMs and clinicopathological features or prognosis. **(A)** M2-TAMs and age; **(B)** M2-TAMs and gender; **(C)** M2-TAMs and pT; **(D)** M2-TAMs and UICC stage; **(E)** M2-TAMs and necrosis; **(F)** M2-TAMs and nuclear grade; **(G)** M2-TAMs and OS; **(H)** M2-TAMs and PFS.
