# Supplementary figures and images for: Prognostic Value of Tumor-Associated Macrophages in Clear Cell Renal Cell Carcinoma: A Systematic Review and Meta-Analysis
Source: Front Oncol. 2021 Apr 26;11:657318. doi: 10.3389/fonc.2021.657318 (PMC8136289; doi:10.3389/fonc.2021.657318)

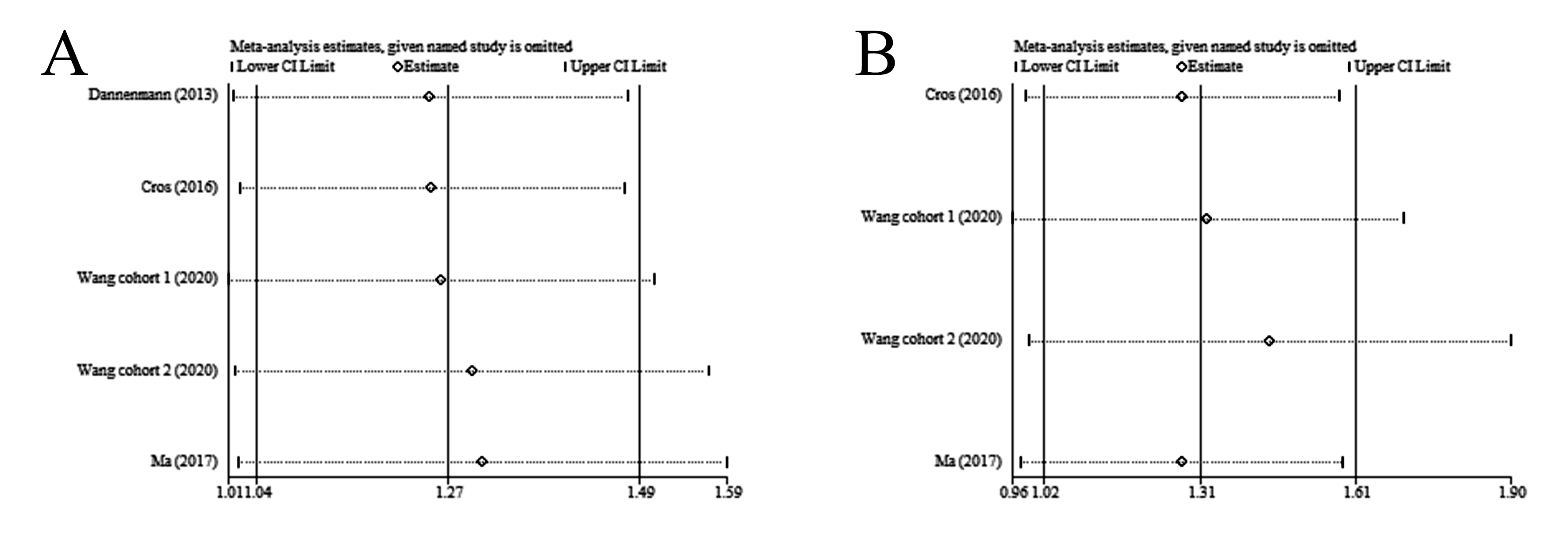

Supplement: Supplementary Figure 1 — Sensitivity analysis. (A) CD163+ TAMs and OS; (B) CD163+ TAMs and PFS. [file Image_1.jpg]

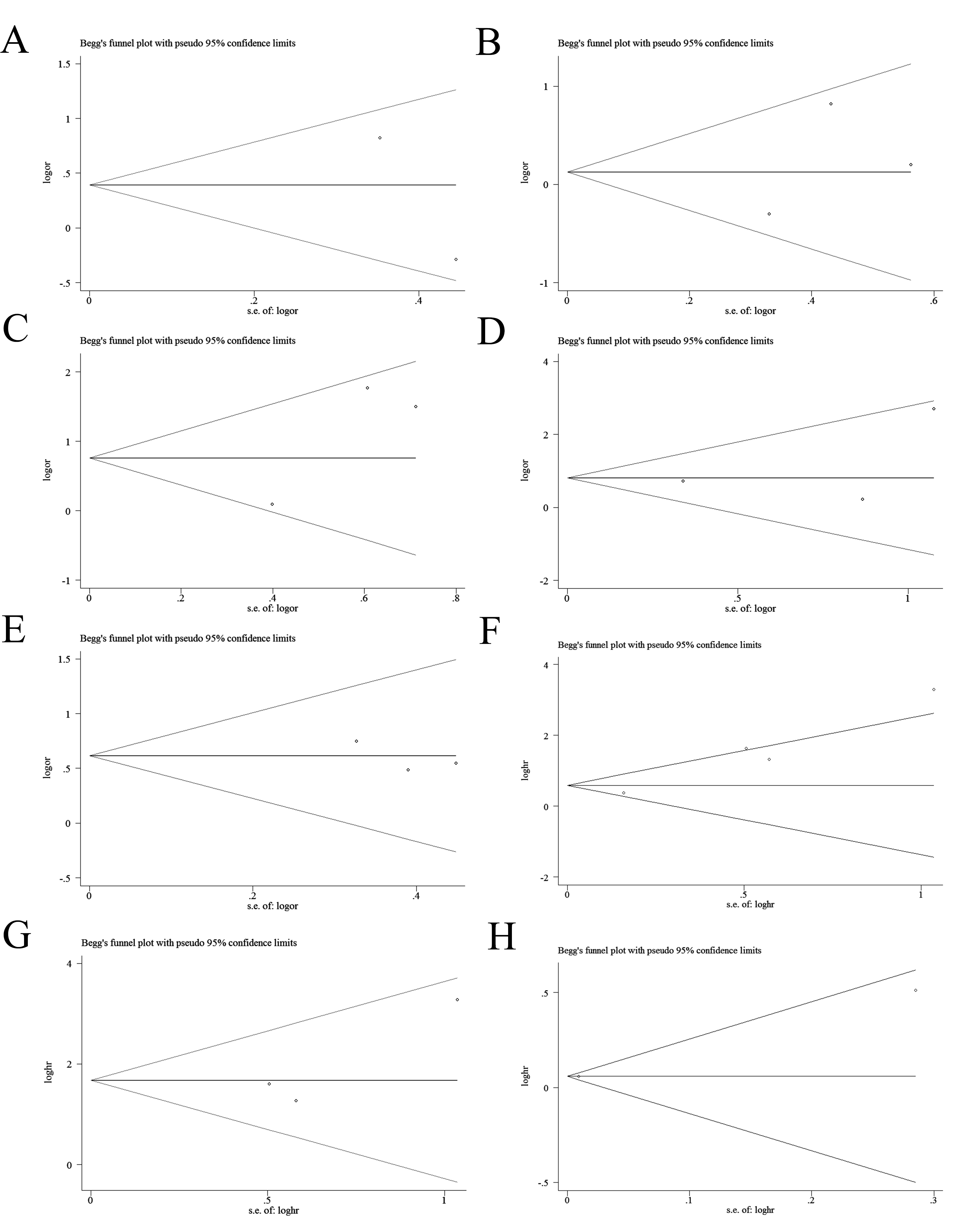

Supplement: Supplementary Figure 2 — The Begg’s funnel plots of the association between CD68+ TAMs and clinicopathological features or prognosis. (A) CD68+ TAMs and age; (B) CD68+ TAMs and gender; (C) CD68+ TAMs and UICC stage; (D) CD68+ TAMs and necrosis; (E) CD68+ TAMs and nuclear grade; (F) CD68+ TAMs and OS; (G) CD68+ TAMs and PFS; (H) CD68+ TAMs and CSS. [file Image_2.jpg]

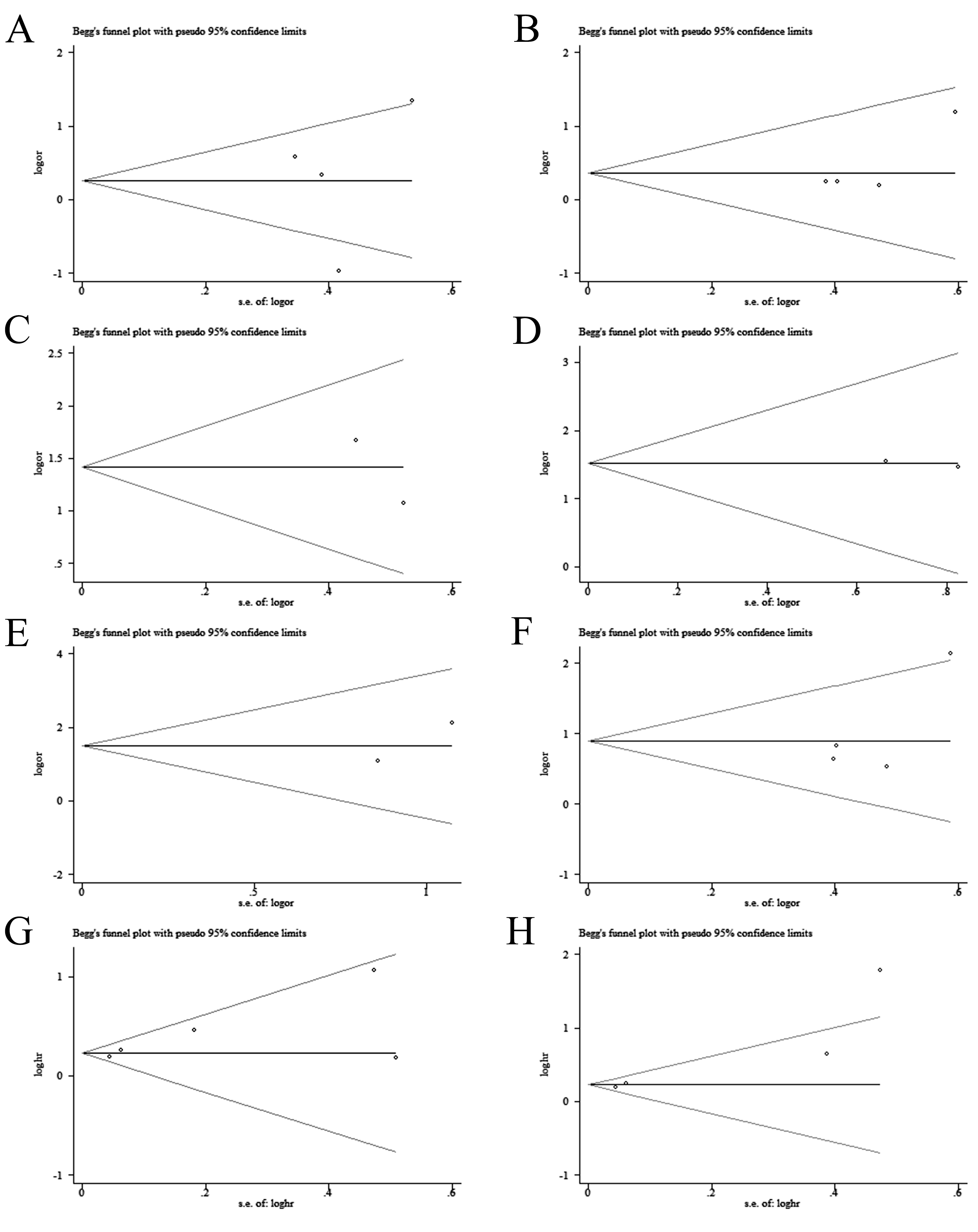

Supplement: Supplementary Figure 3 — The Begg’s funnel plots of the association between CD163+ TAMs and clinicopathological features or prognosis. (A) CD163+ TAMs and age; (B) CD163+ TAMs and gender; (C) CD163+ TAMs and pT; (D) CD163+ TAMs and UICC stage; (E) CD163+ TAMs and necrosis; (F) CD163+ TAMs and nuclear grade; (G) CD163+ TAMs and OS; (H) CD163+ TAMs and PFS. [file Image_3.jpg]

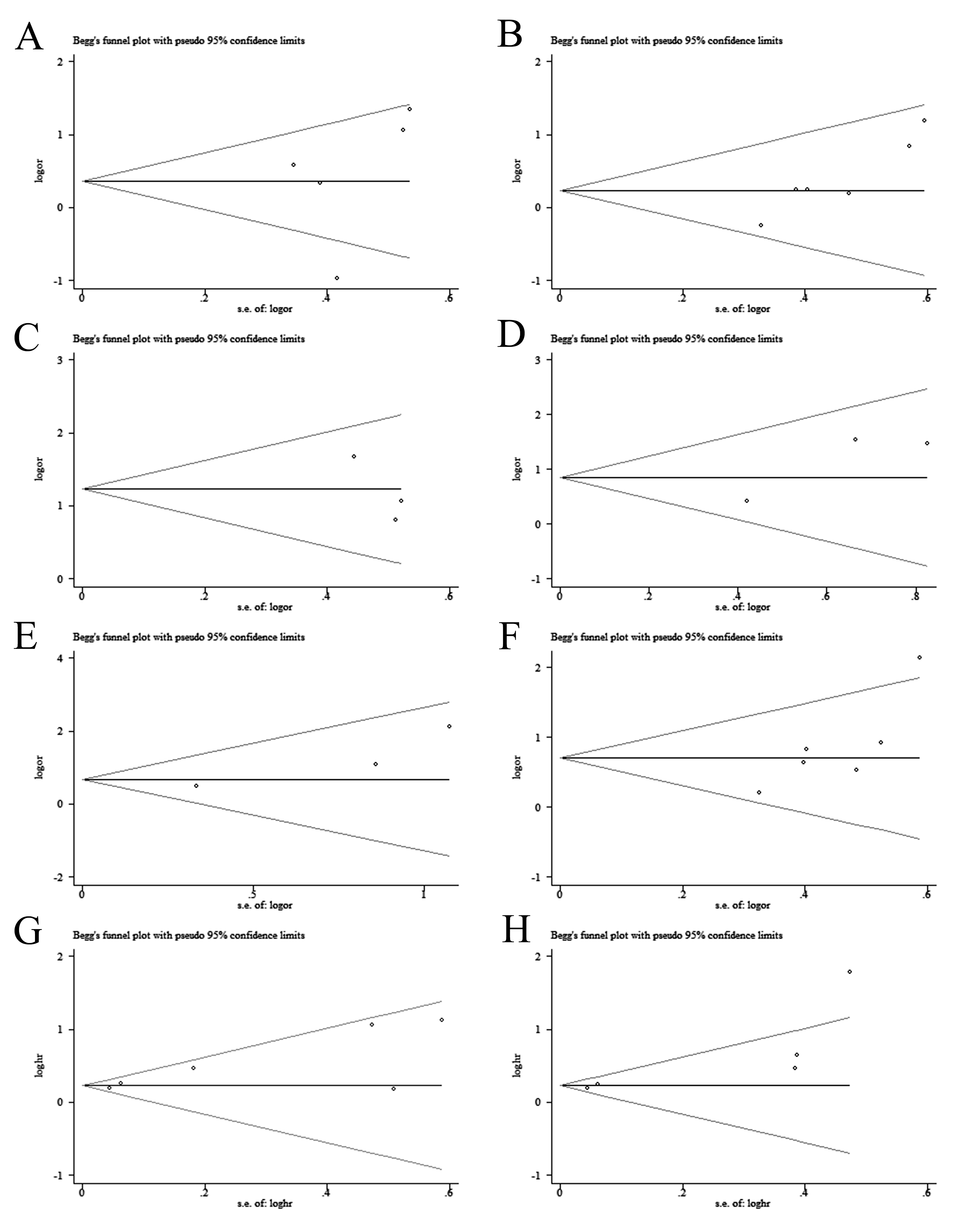

Supplement: Supplementary Figure 4 — The Begg’s funnel plots of the association between M2-TAMs and clinicopathological features or prognosis. (A) M2-TAMs and age; (B) M2-TAMs and gender; (C) M2-TAMs and pT; (D) M2-TAMs and UICC stage; (E) M2-TAMs and necrosis; (F) M2-TAMs and nuclear grade; (G) M2-TAMs and OS; (H) M2-TAMs and PFS. [file Image_4.jpg]
