# Supplementary material for: Prognostic Value of Tumor-Associated Macrophages in Clear Cell Renal Cell Carcinoma: A Systematic Review and Meta-Analysis
Source: Front Oncol. 2021 Apr 26;11:657318. doi: 10.3389/fonc.2021.657318 (PMC8136289; doi:10.3389/fonc.2021.657318)
Supplement: Supplementary file 5 [file Table_1.docx]

**Supplementary Table 1.** Target specimens, assays, dichotomization forms, evaluation methods and cut-off values of TAM detection in the meta-analysis.

| Study | Target specimens | TAM marker and detection assay | Dichotomization form  (high/low density) | Evaluation methods and cut-off values | |
| --- | --- | --- | --- | --- | --- |
| Komohara 2011 | TMA | CD163, CD204  IHC  Samples were reacted with primary antibodies, they were incubated with HRP-labeled goat anti-mouse or anti-rabbit secondary antibodies (Nichirei, Tokyo, Japan). Reactions were visualized using the diaminobenzidine substrate system (Nichirei). | high/low density | The threshold was set as 250 (high, ≥250 cells/mm^2^; low, <250 cells/mm^2^). | |
| Dannenmann 2013 | FFPE tumor tissue | CD68, CD163  Quantitative reverse transcription real-time PCR. | high/low density | Data was dichotomized based on mean gene expression value of all analyzed samples. | |
| Xu 2014 | FFPE tumor tissue | CD68, CD11c, CD206  IHC  EnVision Detection System, Peroxidase/DAB, Rabbit/Mouse (Dako) was used according to the manufacturer’s instructions, with Primary monoclonal antibodies against human CD68 (KP1, 1:500; Dako, Glostrup, Denmark), CD206 (5C11, 1:200; Abcam, Cambridge, MA, USA) and CD11c (EP1347Y, 1:100; Abcam, Cambridge, MA, USA). | high/low density | The cut-off values for low and high CD68, CD206, and CD11c TAM densities were 50 (30th percentile), 30 (30th per-centile), and 55 (80th percentile) cells per field (x200). | |
| Komohara 2015 | FFPE tumor tissue | CD204  IHC  Mouse monoclonal anti-CD204 antibody (SRA-E5; Transgenic) was used for detecting tumor-associated macrophages. | high/low density | The threshold was set as 300 (high, ≥300 cells/mm^2^; low, <300 cells/mm^2^) | |
| Cros 2016 | TMA from FFPE tumor tissue | CD68, CD163  IHC  Mouse monoclonal anti-CD68 (1/200, DAKO Cytomation) and anti-CD163 antibody (1/200, Novocastra) was used for detecting tumor-associated macrophages. | high/low density | CD68 and CD163, global expression was assessed as the score of the three cores. The median expression of each marker within the cohort was used  to separate “high” from “low” expressing tumours. To be qualified as a high-expressing tumour, the mean expression of the cores had to be ≥2 for CD68 and CD163 positive infiltrating macrophages. |  |
| Ma 2017 | FFPE tumor tissue | CD163  IHC  Antibody against CD163 (10D6; Novocastra) was used as primary antibody, and reactions were visualized using the diaminobenzidine substrate system (Nichirei). | high/low density | The threshold was set as 300 (high, ≥300 cells/mm^2^; low, <300 cells/mm^2^) | |
| Nakanishi 2018 | FFPE tumor tissue | CD68  IHC  The anti-CD68 antibody (Leica Bio-systems, New Castle, United Kingdom) was used for IHC. | high/low density | NA. | |
| Wang 2020 cohort 1 | FFPE tumor tissue | CD68, CD163  IHC  The following primary antibodies were used: mouse anti-CD68 (1:200, ab53444, Abcam), rabbit anti-CD163 antibody (1:100, ab189915, Abcam). Three randomly representative areas of the tumor were evaluated at ×200  magnification to score the density of stained stromal immune cells. Finally, the mean value was calculated. The total cell count was defined as the number of nucleated stained cells per field and is presented as the density (cells/mm^2^). | high/low density | The optimal cut-off value of CD68 and CD163 for dividing ccRCC patients was determined by a time-dependent ROC analysis. | |
| Wang 2020 cohort 2 | FFPE tumor tissue | CD68, CD163  IHC  The following primary antibodies were used: mouse anti-CD68 (1:200, ab53444, Abcam), rabbit anti-CD163 antibody (1:100, ab189915, Abcam). Three randomly representative areas of the tumor were evaluated at ×200  magnification to score the density of stained stromal immune cells. Finally, the mean value was calculated. The total cell count was defined as the number of nucleated stained cells per field and is presented as the density (cells/mm^2^). | high/low density | The optimal cut-off value of CD68 and CD163 for dividing ccRCC patients was determined by a time-dependent ROC analysis. | |

FFPE, formalin-fixed paraffin-embedded; TMA, tissue microarrays; IHC, immunohistochemistry; TAM, tumor-associated macrophage; ccRCC, clear cell renal cell carcinoma;

ROC, receiver operating characteristic; NA, not available.

| Source | Selection | | | | Comparability  Comparability  of cohorts on the basis of the design or analysis | Outcome | | | Overall |
| --- | --- | --- | --- | --- | --- | --- | --- | --- | --- |
| Study | Representativeness of exposed cohort | Selection of non-exposed cohort | Ascertainment of exposure to implants | Outcome not present at start |  | Assessment of outcome | Adequate follow-up length | Adequacy of follow-up |  |
| Komohara 2011 | ★ | ★ | ★ |  | ★★ | ★ | ★ |  | 7 |
| Dannenmann 2013 | ★ | ★ | ★ |  | ★ | ★ | ★ | ★ | 7 |
| Xu 2014 | ★ | ★ | ★ |  | ★★ | ★ | ★ | ★ | 8 |
| Komohara 2015 | ★ | ★ | ★ |  | ★★ | ★ | ★ |  | 7 |
| Cros 2016 | ★ | ★ | ★ |  | ★★ | ★ | ★ | ★ | 8 |
| Nakanishi 2018 | ★ | ★ | ★ |  | ★ | ★ | ★ | ★ | 7 |
| Ma 2017 | ★ | ★ | ★ |  | ★★ | ★ | ★ |  | 7 |
| Wang 2020 | ★ | ★ | ★ |  | ★★ | ★ | ★ |  | 7 |

**Supplementary Table 2.** Newcastle-Ottawa scale quality scores of the included studies.

| Group | No. of studies | Chi² | *p*_heterogeneity_ | I² (%) | Pooled OR/HR (95% CI) | | | | Begg's test |
| --- | --- | --- | --- | --- | --- | --- | --- | --- | --- |
|  |  |  |  |  | Fixed model | *p* value | Random model | *p* value | *p* value |
| CD68+ TAM Age (>60 VS ≤60) | 1 | 3.83 | 0.05 | 73.9 | 1.47 (0.86-2.52) | 0.156 | 1.35 (0.45-3.99) | 0.592 | 0.317 |
| Gender (male VS female) | 2 | 4.27 | 0.118 | 53.2 | 1.16 (0.73-1.83) | 0.528 | 1.22 (0.60-2.49) | 0.589 | 0.602 |
| nuclear grade (G3,4 VS G1,2) | 2 | 0.29 | 0.864 | 0.0 | 1.85 (1.21-2.84) | 0.005 | 1.85 (1.21-2.84) | 0.005 | 0.602 |
| Tumor necrosis (+ VS -) | 2 | 3.70 | 0.157 | 46.0 | 2.47 (1.39-4.37) | 0.002 | 2.64 (0.88-7.92) | 0.084 | 0.602 |
| UICC stage (III, IV VS I, II) | 2 | 6.63 | 0.036 | 69.8 | 2.14 (1.19-3.85) | 0.011 | 2.80 (0.88-8.96) | 0.083 | 0.602 |
| OS (CD68+ TAM high VS low) | 3 | 14.62 | 0.002 | 79.5 | 1.81 (1.36-2.41) | <0.001 | 3.97 (1.39-11.39) | 0.010 | 0.174 |
| CSS (CD68+ TAM high VS low) | 2 | 2.52 | 0.112 | 60.3 | 1.06 (1.04-1.08) | <0.001 | 1.22 (0.81-1.83) | 0.348 | 0.317 |
| PFS (CD68+ TAM high VS low) | 2 | 2.90 | 0.234 | 31.1 | 5.35 (2.65-10.78) | <0.001 | 5.73 (2.36-13.90) | <0.001 | 0.602 |

**Supplementary Table 3.** Meta-analysis results for the clinicopathological significance and prognostic value of CD68+ TAM in ccRCC

| Group | No. of studies | Chi² | *p*_heterogeneity_ | I² (%) | Pooled OR/HR (95% CI) | | | | Begg's test |
| --- | --- | --- | --- | --- | --- | --- | --- | --- | --- |
|  |  |  |  |  | Fixed model | *p* value | Random model | *p* value | *p* value |
| Age (>60 VS ≤60) | 3 | 13.62 | 0.003 | 78.0 | 1.29 (0.88-1.89) | 0.201 | 1.35 (0.57-3.19) | 0.501 | 1.000 |
| Gender (male VS female) | 3 | 2.23 | 0.527 | 0.0 | 1.46 (0.94-2.24) | 0.089 | 1.44 (0.93-2.23) | 0.102 | 0.497 |
| nuclear grade (G3,4 VS G1,2) | 3 | 5.45 | 0.142 | 44.9 | 2.48 (1.61-3.83) | 0.000 | 2.57 (1.40-4.72) | 0.002 | 0.497 |
| Tumor necrosis (+ VS -) | 1 | 0.56 | 0.453 | 0.0 | 4.82 (1.33-17.51) | 0.017 | 4.43 (1.19-16.52) | 0.027 | 0.317 |
| UICC stage (III, IV VS I, II) | 1 | 0.00 | 0.945 | 0.0 | 4.56 (1.62-12.58) | 0.003 | 4.55 (1.65-12.57) | 0.003 | 0.317 |
| OS (CD163+ TAM high VS low) | 4 | 5.64 | 0.227 | 29.1 | 1.26 (1.17-1.35) | <0.001 | 1.29 (1.15-1.44) | <0.001 | 0.327 |
| PFS (CD163+ TAM high VS low) | 3 | 12.52 | 0.006 | 76.0 | 1.26 (1.17-1.35) | <0.001 | 1.40 (1.12-1.75) | 0.003 | 0.089 |

**Supplementary Table 4.** Meta-analysis results for the clinicopathological significance and prognostic value of CD163+ TAM in ccRCC.

| Group | No. of studies | Chi² | *p*_heterogeneity_ | I² (%) | Pooled OR (95% CI) | | | | Begg's test |
| --- | --- | --- | --- | --- | --- | --- | --- | --- | --- |
|  |  |  |  |  | Fixed model | *p* value | Random model | *p* value | *p* value |
| Age (>60 VS ≤60) | 3 | 15.66 | 0.004 | 74.5 | 1.42 (0.99-2.04) | 0.055 | 1.54 (0.73-3.26) | 0.258 | 0.327 |
| Gender (male VS female) | 3 | 5.79 | 0.327 | 13.7 | 1.28 (0.91-1.80) | 0.149 | 1.30 (0.89-1.89) | 0.173 | 0.060 |
| nuclear grade (G3,4 VS G1,2) | 4 | 8.60 | 0.126 | 41.9 | 2.04 (1.46-2.86) | 0.000 | 2.16 (1.36-3.43) | 0.001 | 0.060 |
| Tumor necrosis (+ VS -) | 2 | 2.39 | 0.303 | 16.3 | 2.12 (1.20-3.75) | 0.010 | 2.20 (1.03-4.71) | 0.042 | 0.117 |
| UICC stage (III, IV VS I, II) | 2 | 2.68 | 0.261 | 25.5 | 2.44 (1.30-4.59) | 0.005 | 2.58 (1.18-5.65) | 0.018 | 0.602 |
| OS (CD68+ TAM high VS low) | 5 | 8.00 | 0.156 | 37.5 | 1.26 (1.18-1.35) | <0.001 | 1.32 (1.16-1.50) | <0.001 | 0.188 |
| PFS (CD68+ TAM high VS low) | 4 | 12.92 | 0.012 | 69.0 | 1.26 (1.17-1.35) | <0.001 | 1.40 (1.14-1.72) | 0.001 | 0.270 |

**Supplementary Table 5.** Meta-analysis results for the clinicopathological significance and prognostic value of M2-TAM in ccRCC.
